# Development of a mouse model for the visual and quantitative assessment of lymphatic trafficking and function by *in vivo* imaging

**DOI:** 10.1038/s41598-018-23693-9

**Published:** 2018-04-12

**Authors:** Yoshihisa Yamaji, Shinsuke Akita, Hidetaka Akita, Naoya Miura, Masaki Gomi, Ichiro Manabe, Yoshitaka Kubota, Nobuyuki Mitsukawa

**Affiliations:** 1 Department of Plastic, Reconstructive, and Aesthetic Surgery, Chiba University Graduate School of Medicine, Chiba, 2608677 Japan; 2 Laboratory of Pharmacology and Toxicology, Chiba University Graduate School of Pharmaceutical Sciences, Chiba, 2608677 Japan; 3 Department of Aging Research, Chiba University Graduate School of Medicine, Chiba, 2608677 Japan

## Abstract

Methods for quantitative analysis of long distance lymphatic transport of nanoparticles in live animals are yet to be established. We established a mouse model for analysis of time-dependent transport just beneath the abdominal skin to investigate lymph node-to-lymph node trafficking by *in vivo* imaging. For this purpose, popliteal lymph nodes (PLNs) as well as efferent and afferent lymphatic vessels, marginal veins, and feeding blood vessels were surgically resected to change the lymphatic flow from footpad injections. Using this model, we observed a novel lymphatic flow from the footpad to the proper axillary lymph node (ALN) via the inguinal lymph node (ILN). This drainage pathway was maintained over 12 weeks. Time-dependent transportation of 1,1′-dioctadecyltetramethyl indotricarbocyanine iodide-labelled liposomes from the footpad to the ILN was successfully quantified by an *in vivo* imaging system. Moreover, congestion and development of a new collateral lymphatic route was visualised under a lymphedema status. Histological analysis of abdominal skin tissues of this model revealed that PLN resection had no effect on the abdominal lymphatic system between the ILN and ALN. These data indicate that this model might be useful to clarify the mechanisms of lymphedema and study direct transportation of lymph or other substances between lymph nodes.

## Introduction

The lymphatic system has essential functions in cancer progression, lymphedema, and chronic inflammation^1,2^. Despite the appeal of lymphatic targeting therapies, an ideal animal model to investigate lymphatic transport of drugs and cells (metastatic cancer), and lymphatic flow in lymphedema is yet to be established. As an ideal experimental model, the lymphatic system must be visible non-invasively and quantitatively over time.

Advances in lymphatic imaging methods based on fluorescence in the near infrared region (NIR) have progressed in the last decade^3,4^. Indocyanine green (ICG) is a typical NIR dye widely used in humans^3^. However, because of its low molecular weight, it easily leaks into the blood circulation from injection sites, collecting in lymphatic vessels or high endothelial venules of lymph nodes (LNs)^5^. Nano-sized liposomes loaded with fluorophores are a useful probe for quantitative evaluation of lymphatic flow because liposomes of <100 nm in size preferentially drain into the lymph nodes^6^ and are retained in lymphatic vessels. Furthermore, liposomes are a promising tool for drug delivery. Thus, control of the lymphatic transport of liposomes through the lymphatic system may enable novel therapeutic approaches for lymphedema. Therefore, methods for kinetic analysis of long distance lymphatic vessels, especially via LNs, is highly desired.

**Figure 1:**
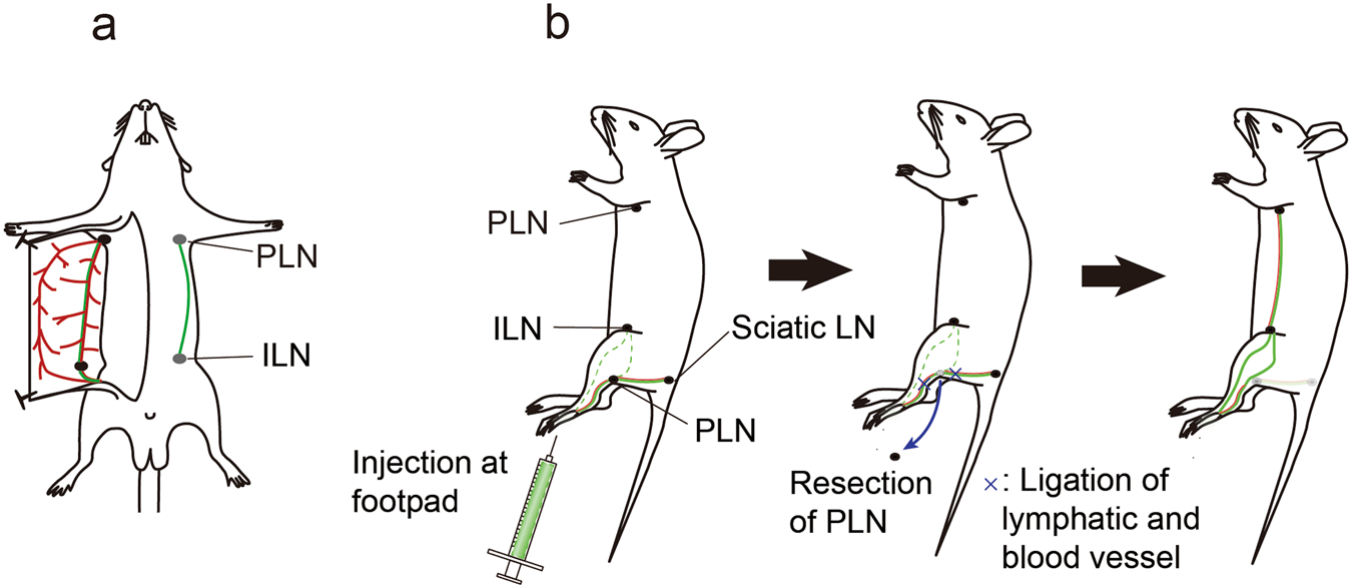
Schematic diagram illustrating a lymphatic flow-modified mouse. (**a**) Definition of the inguinal lymph node (ILN) to proper axillary lymph node (ALN). (**b**) Procedure to establish a lymphatic flow-modified mouse.

Mice have 22 LNs and peripheral inter-LN vessels^7^. Among these vessels, the lymphatic vessel from the inguinal LN (ILN) to the proper axillary LN (ALN) has superior accessibility for surgical procedures and ease of observation (Fig. 1a). In addition, it traverses a long distance with one-way flow to the ALN via the ILN with no branching networks. Because of these features, the lymphatic system from the ILN to ALN area was selected to investigate lymphatic system mechanisms^8–12^.

The mouse hind footpad is a convenient and widely used injection site of various substances^13–15^. However, it was reported that a solution injected in the hind footpad dominantly drains to the popliteal LN (PLN), then to the ischial LN, and lumbar LN^16^. Because this route flows deep under muscle, non-invasive quantitative analysis is difficult.

Kwon *et al*. reported^17^ that the drainage pattern changes dramatically after PLN resection, and all injected fluorescence dye (ICG) injected to the footpad drains to the ILN. However, this modified lymphatic route is temporary because the route of lymphatic flow from the paw to the ischial LN via the resected PLN region recovers after approximately 20 days. To evaluate the lymphatic flow in lymphedema, a mouse model that retains the ILN-directed lymphatic flow for a longer period is necessary because induction of lymphoedematous conditions generally requires at least 12 weeks^18^. Furthermore, such a mouse model might allow quantitative analysis of lymphatic transport of nanoparticles with high reproducibility.

In this study, we developed an advanced surgical method for resection of the PLN to induce ILN-directed lymphatic flow for at least 12 weeks. This model is useful for quantitative analysis of long distance ILN-mediated transport of liposomal nanoparticles towards the ALN using an *in vivo* imaging system.

## Results

**Figure 2:**
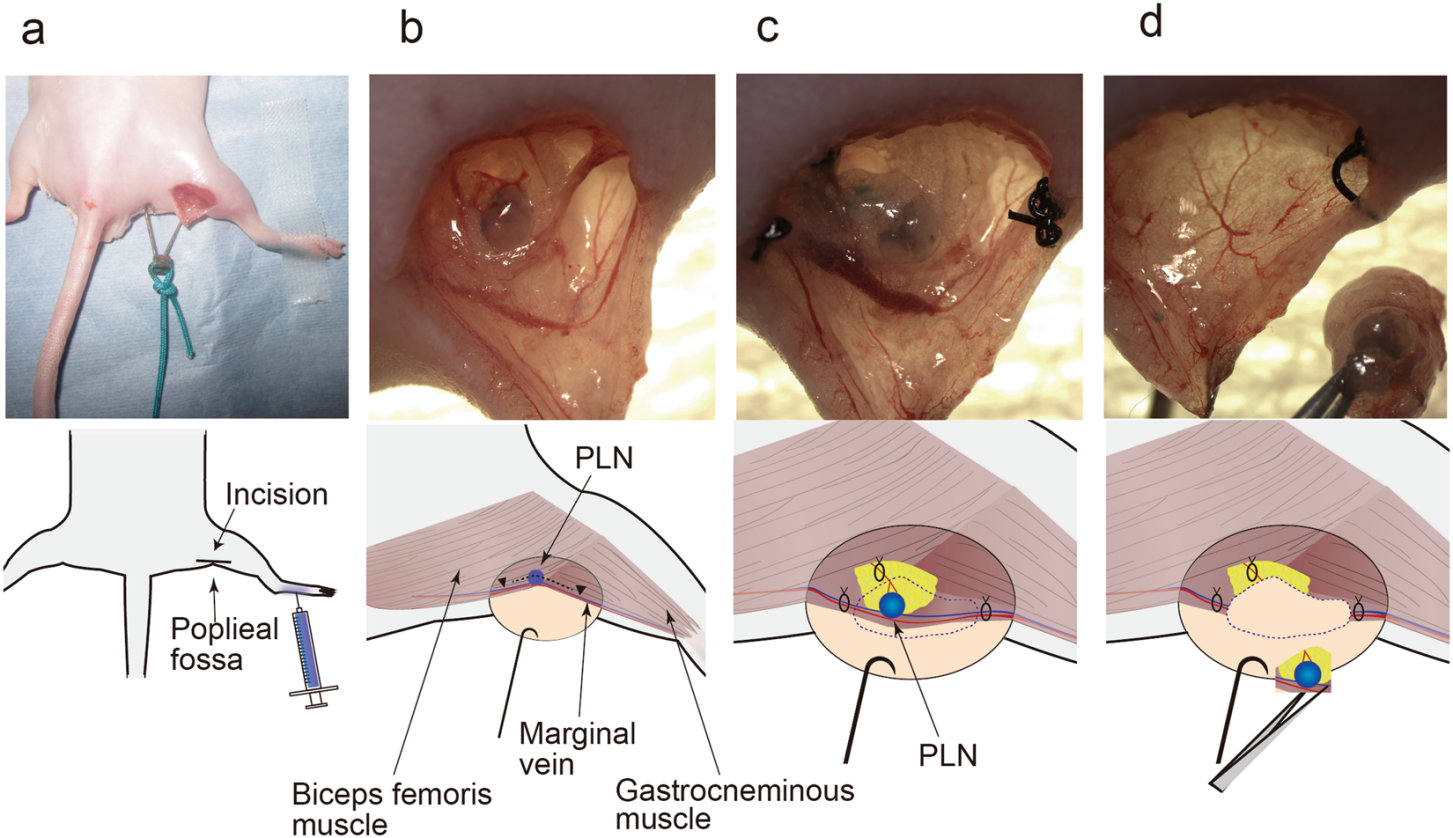
Representative intravital image for the resection procedure of the PLN. Upper panels: images; lower panels: schema. (**a**) After Evans blue dye (EBD) injection into the left footpad, a 10 mm longitudinal incision was made at the popliteal fossa. (**b**) The PLN under the biceps femoris muscle was exposed with the aid of EBD staining. Black arrowhead; lymphatic vessel along the marginal vein. (**c**) Efferent and afferent lymphatic vessels, marginal vein, and blood vessels towards the PLN were ligated. (**d**) The PLN with connecting blood and lymphatic vessels were removed.

**Figure 3:**
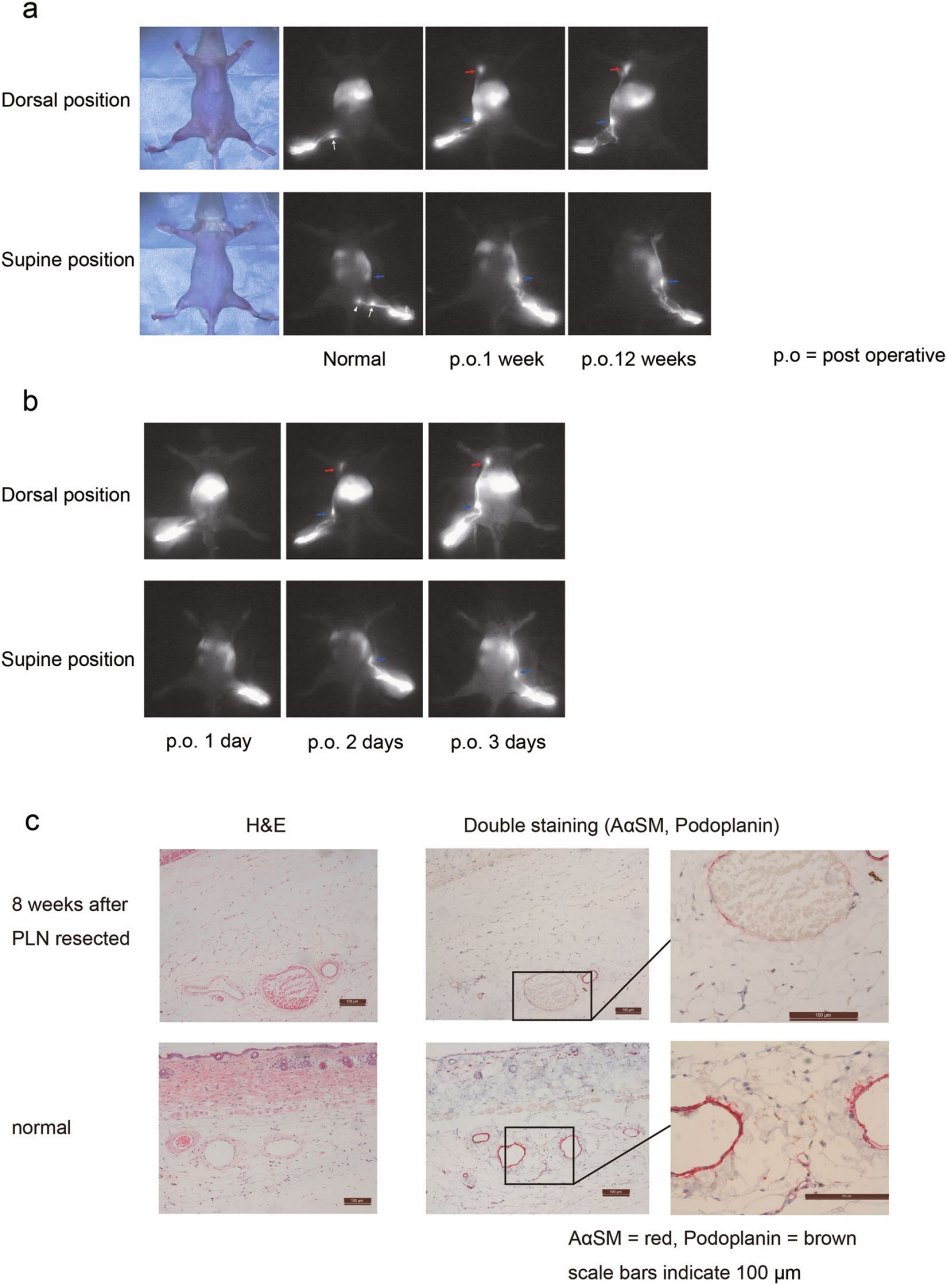
Typical image of the lymphatic flow in a normal mouse and PLN-resected mouse. ICG was injected into the footpad in (**a**) normal and PLN-resected mice (1 and 12 weeks after resection), and (**b**) PLN-resected mice (1–3 days after resection), followed by visualisation using the PDE. White, blue, and red arrows represent the PLN, ILN and ALN, respectively. The white arrowhead represents the ischial LN. (**c**) Immunohistochemical analysis showing the effect of PLN resection on the lymphatic vessel configuration in abdominal skin.

A lymph flow-modified mouse was established by PLN resection (Fig. 1b). The surgical procedure is shown in Fig. 2. Development of a novel ILN-mediated lymphatic flow pathway from the footpad to the ALN was confirmed at 1 week after resection of the PLN. At 1 h after footpad injection of ICG, lymphatic flow was visualised by a near infrared video camera system (Photodynamic Eye, PDE; Hamamatsu Photonics, Hamamatsu, Japan). Typical images are shown in Fig. 3a. In five normal mice, ICG flowed predominantly into the PLN, and fluorescent signals were not detected in the ALN or ILN. Of note, a long lymphatic route was not detected, although the flow to the ischial LN via the PLN was visible. This result confirmed the difficulty in observing lymphatic flow when it drains deep into tissues (i.e. the lumbar LN). In contrast, at 1 week after PLN resection, a long lymphatic flow to the ALN via the ILN was clearly visible in all 10 mice analysed. The route towards the PLN-resected region and further flow to the ischial LN was not detected in any mice (Fig. 3a). The most significant finding was that the modified lymphatic flow towards the ALN had remained at 12 weeks after PLN resection in all mice (n = 5).

To establish the PLN-resected mouse as an experimental model to visualise lymph-mediated transport and/or perturbation of the lymphatic system under pathological conditions, the minimum number of days necessary for this change of lymphatic flow were determined (Fig. 3b). At 1 day after PLN resection, lymphatic flow into the ILN was poor. In contrast, lymphatic flow to the ILN was obvious at day 2 and more clearly detected at day 3. Therefore, the following experiments were performed at least 4 days after PLN resection.

To investigate whether PLN resection affected the abdominal lymphatic system, histological analysis of abdominal skin tissue was performed. Histological changes were not observed at 8 weeks after PLN resection compared with the abdominal skin of a normal mouse (Fig. 3c) indicating that an increase in the afferent route to the ILN had no effect on the efferent lymphatic system to the ALN.

**Figure 4:**
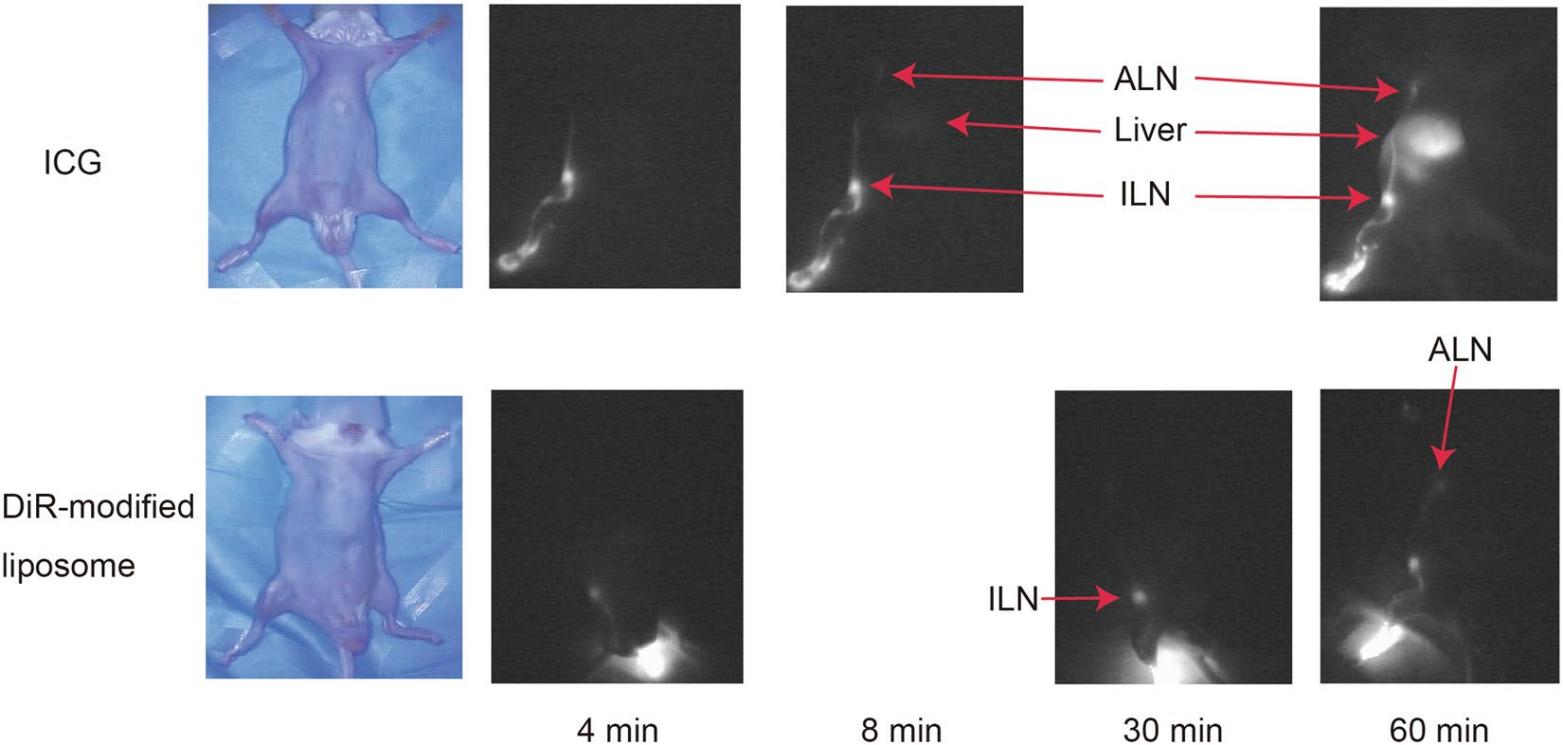
Imaging of the lymphatic transport of ICG and DiR-labelled liposomes. ICG and DiR-labelled liposomes were injected into the footpad of PLN-resected mice (day 7) and visualised by the PDE at the indicated times.

Next, we compared the difference in speed of lymphatic drainage of a low molecular compound (ICG) and nanoparticles [1,1′-dioctadecyltetramethyl indotricarbocyanine iodide (DiR)-labelled liposomes]. In all ICG-injected mice, fluorescence in the ALN along with the lymphatic duct were detectable within 8 min. Of note, the fluorescent signal was obvious in the liver within 10 min after footpad injection, and it became stronger after 60 min. In contrast, for DiR-modified liposomes, fluorescent signals were first detected in the ILN at 30 min and then in the ALN at 1 h after injection. Fluorescence was not detected in the liver by 60 min after injection (Fig. 4).

**Figure 5:**
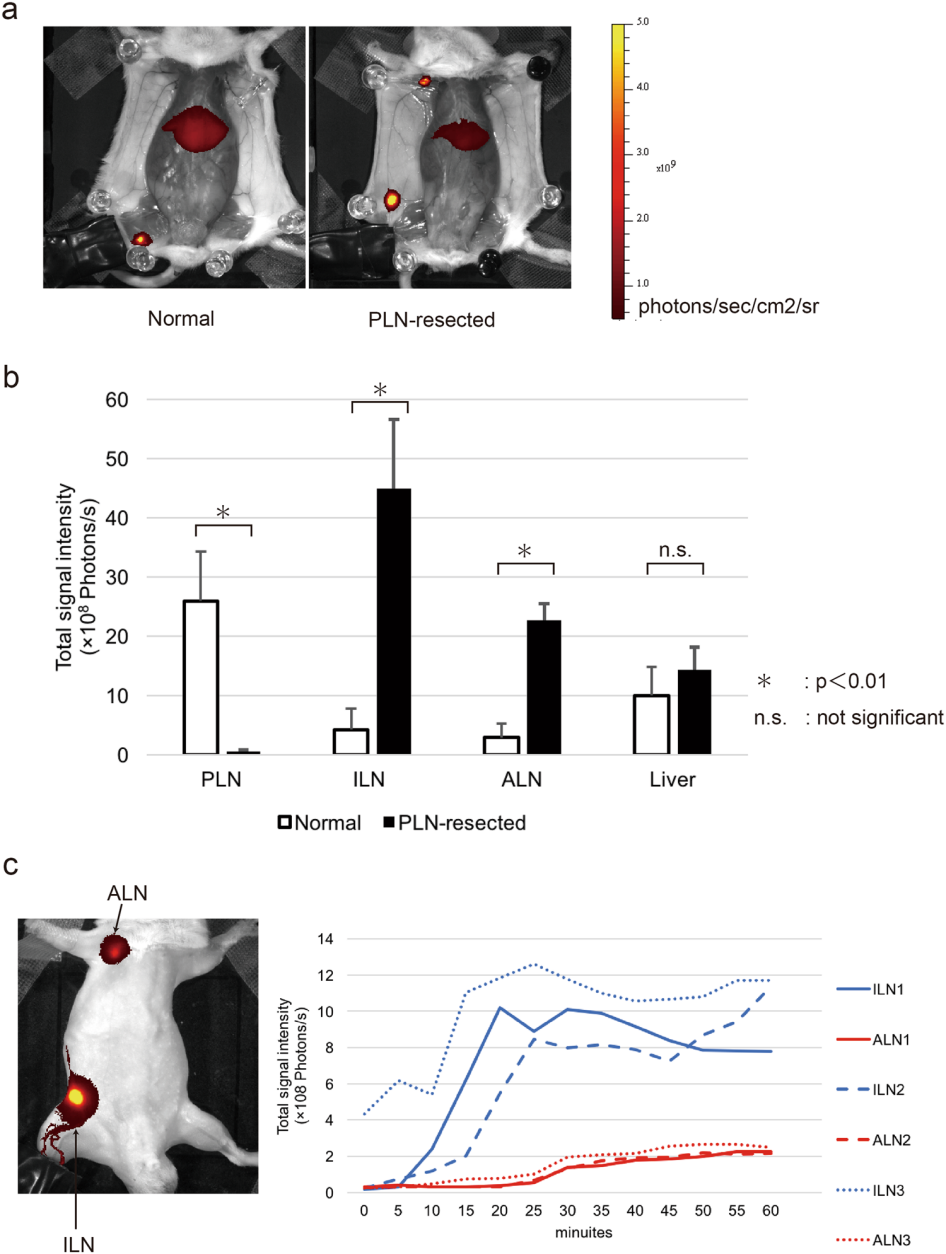
Quantitative analysis of DiR-labelled liposomes in PLN-resected mice using the IVIS. (**a**) Typical images of the distribution of DiR-labelled liposomes. (**b**) Quantification of the fluorescent signals derived from DiR-labelled liposomes in the PLN, ILN, ALN, and liver of normal and PLN-resected mice at 120 min after the injection of DiR-modified liposomes. (**c**) Time-dependent change of fluorescent signals in the ILN and ALN.

To demonstrate the utility of the PLN-resected mouse for quantitative analysis of LN-to-LN transport, accumulation of fluorescent signals derived from DiR-labelled liposomes in the PLN, ILN, and ALN was quantified by a non-invasive fluorescence *in vivo* imaging system (IVIS, Lumina II; Caliper Life Science, Hopkinton, MA, USA) in normal and PLN-resected mice at 2 h after injection. Consistent with the result of ICG transport (Fig. 3), accumulation of DiR-labelled liposomes was predominantly observed in the PLN of normal mice, whereas fluorescent signals were highly detected in the ILN and ALN of PLN-resected mice (Fig. 5a). Quantitative analysis of four mice (Fig. 5b) revealed that the accumulation of liposomes in the ILN of PLN-resected mice (44.81 ± 11.75) was significantly higher than that in normal mice (4.22 ± 3.58) (P < 0.01). Similarly, accumulation in the ALN of PLN-resected mice (22.61 ± 2.81) was higher than in normal mice (2.88 ± 2.33) (P < 0.01). In contrast, hepatic accumulation was comparable between PLN-resected mice (14.35 ± 3.8) and normal mice (9.96 ± 4.82) (Fig. 5a and b). Thus, in mice whose lymphatic route was changed by PLN resection, the ability of lymphatic transportation from the footpad to the blood circulation system at the venous angle was not markedly compromised compared with mice with a normal lymphatic system. The time profile for liposomal accumulation in the ILN and ALN was monitored in PLN-resected mice (day 7) (n = 3). As shown in Fig. 5c, fluorescence in the ILN increased rapidly to the maximum level within 25 min. At 30 min after injection, the corresponding value decreased gradually in parallel with an increase of fluorescence in the ALN. This observation indicates a reasonable mass balance in terms of quantification of the lymphatic transport from the ILN to ALN (Fig. 5c).

**Figure 6:**
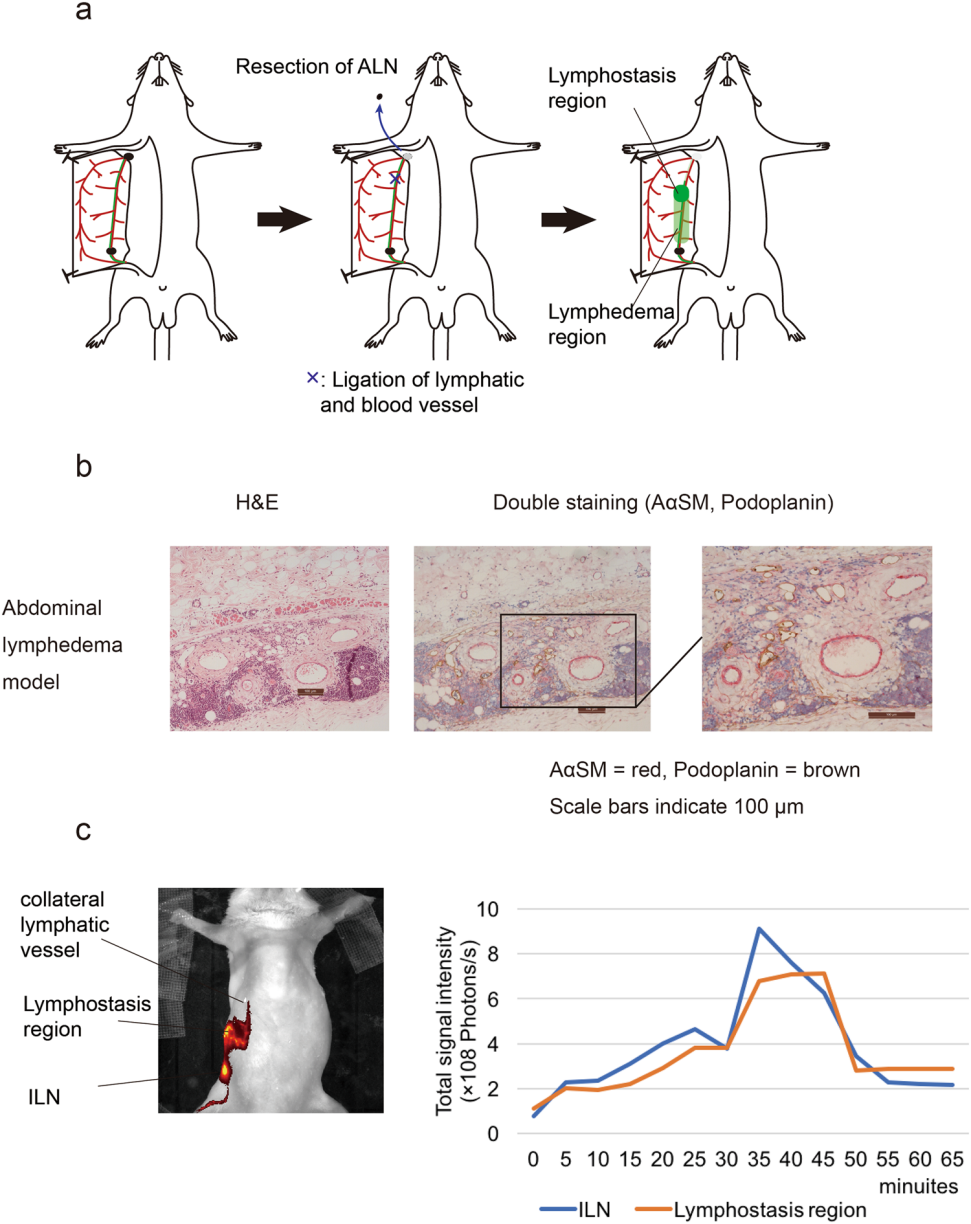
Quantification of the accumulation of DiR-labelled liposomes in the lymphostatic model. (**a**) Schematic diagram of the procedure used to establish an abdominal lymphedema model by resecting the ALN and ligating the lymphatic vessel toward the ALN. (**b**) Histological analysis showing development of lymph vessel hyperplasia in the lymphostatic model. (**c**) Quantification of fluorescent signals derived from DiR-labelled liposomes in the ILN and lymphostasis area.

To analyse the change in lymphatic flow under lymphostasis, an abdominal wall lymphedema model was established. In this model, the tail side region just below the ligation site was defined as the lymphostasis region, and the area between the ligation site and ILN was defined as the lymphedema region (Fig. 6a).

To confirm abdominal wall lymphedema, histological analysis of abdominal skin tissue was performed. The presence of prominent fibrotic changes together with hyperplasia of lymph vessels in subdermal tissues indicated that the area between the ligation site and ILN was under a lymphedema status.

The fluorescence intensity in the lymphostasis region of interest (ROI) was increased temporarily and then decreased. This observation suggested that the lymphostasis might have triggered the formation of a new collateral route (Fig. 6c).

## Discussion

Imaging is an important technology to analyse lymphatic transport and dynamics of lymphatic vessels. In normal mice, the efferent lymphatic flow from the footpad is predominantly directed into the PLN, followed by the ischial LN and lumbar LN located deeply under muscles^14,16^. This conventional lymphatic route hampers quantitative analysis of LN-to-LN transport, especially when using an IVIS. In this study, we successfully established a mouse model of long distance lymphatic flow toward the ALN via the ILN just beneath the surface of the abdominal skin, which remained for at least for 12 weeks. Kwon *et al*.^12^ and Robinson *et al*.^19^ assessed the lymphatic propagation velocity and frequency of lymphatic propulsion in abdominal lymphatic vessels by injection of fluorophores at the base of the mouse tail. In this case, the authors emphasised that intradermal rather than subcutaneous injection was important, because subcutaneous injection resulted in unstable uptake into the lymphatic flow. Thus, a more convenient method to allow injected molecules to drain into the lymphatic system is necessary. In PLN-resected mice, substances injected into the footpad stably reached the ILN within 30 min after injection with high reproducibility. The histological characteristics of footpad tissues, a thin subcutaneous layer and thick skin, are considered as advantageous for uptake of molecules into the lymphatic system.

Two minor routes of lymphatic flow to the ILN after footpad injection have been reported previously, namely direct and indirect routed via the PLN^15,20,21^. However, our quantitative analysis revealed that fluorescent signals in the ILN were similar to background levels in normal mice, even when accumulation of DiR-labelled liposomes was clearly detected in the PLN (Fig. 5b). Thus, in the mice used in our study, PLN-mediated lymphatic flow to the ILN was negligible.

Kwon *et al*. reported^17^ that the drainage pattern changes dramatically after PLN resection. A fluorescent dye injected into the mouse footpad temporarily drained to the ILN. However, lymphatic flow to the PLN was recovered within 4 weeks, presumably because of lymphangiogenesis. Hoshi *et al*.^14^ reported that the afferent lymphatic flow is important for the growth of lymph nodes, because lymphoid organisation of the PLN was perturbed for 15 weeks when the afferent lymphatic flow to this node was blocked by resection of lymphatic vessels. In their report, the authors also resected a surrounding vein and fascia, as well as lymphatic vessels. Based on these previous studies, we resected the PLN along with surrounding vessels to completely prevent lymphomagenesis at the resected site. This procedure might explain the successful induction of a stable lymphatic route towards the ILN without regeneration of the lymph flow to the PLN for at least 12 weeks after the operation (Fig. 3a). In general, lymphangiogenesis and/or collateral bypass is more likely to occur under lymphostatic conditions^17,18,22^ as shown in the abdominal lymphedema model (Fig. 6).

While the mechanism remains to be clarified, platelet-derived growth factor (PDGF) might be one of the factors that induce lymphangiogenesis^23^. Because endothelial cells and platelets are sources of PDGF, the ligation and resection of blood vessels surrounding the PLN might prohibit the regeneration of lymphatic flow in the resected region.

The short-term change in the drainage pattern (3 days after PLN resection; Fig. 3b) suggested that pre-existing lymphatic vessels whose functions were conventional in the resting state, rather than the newly developed vessels by lymphangiogenesis, contributed to the ILN-directed lymphatic flow in the hindlimbs of PLN-resected mice. The hydraulic pressure caused by the temporal congestion of lymphatic flow by resection of the PLN triggered drainage into the collateral lymphatic vessel.

Liposomal formulation of low molecular weight fluorophores has great advantages to enhance fluorescent signals by their clustering effect. It also improves their uptake and retention in the lymphatic system^5^. The optimal particle size for a tracer of the sentinel LN in relation to cancer metastasis has been studied previously^24–27^. In general, liposomes with particle sizes of <100 nm are taken up into lymphatic capillaries^28–31^. Based on this information, we used a liposomal particle size of approximately 100 nm as a probe for lymphatic transport. Consistent with the previous reports, this size of DiR-labelled liposomes rapidly entered the lymphatic system and ILN within 20 min and then readily flowed into the ALN. The effect of the particle size and ζ-potentials of ILN-mediated lymphatic transport toward the ALN is currently being studied.

Because this model is simple to establish, it will be useful for research on cancer metastasis beyond the sentinel LN, and lymphangiogenesis and/or inflammation in oedematous states^18,32^. It might also be useful for quantitative evaluation of the efficiency of lymphatic drug delivery.

## Conclusion

A simple mouse model was established to observe the LN-mediated lymphatic flow towards the next LN. This mouse model allowed quantitative analysis of the transport of nanoparticles using an IVIS. Furthermore, this mouse model allowed visualisation of lymphostasis. Therefore, this model might contribute to investigation of the onset mechanisms in relation to lymphedema and comparisons of the therapeutic efficacy of various surgery treatments or drug delivery in future studies.

## Material and Methods

### Experimental animal models

We used 8-week-old male ICR mice purchased from Takasugi Experimental Animals Supply Corporation (Saitama, Japan). All of the following research protocols including the surgical procedures and animal care were approved by the Institutional Animal Care and Use Committee of Chiba University (approval ID: 29–79), and all methods were performed in accordance with the relevant guidelines and regulations.

The procedure for establishment of a lymph flow-modified mouse is shown in Fig. 2. Mice were anesthetised with isoflurane. Then, 0.02 ml of 5 mg/ml Evans blue dye (EBD) solution was injected into their left footpads. A 10 mm longitudinal incision was made at the popliteal fossa (Fig. 2a) to expose a PLN that was stained blue by the injection of EBD under the biceps femoris muscle (Fig. 2b). Then, the PLN, efferent and afferent lymphatic vessels, marginal vein, and feeding blood vessels were completely resected after tight ligation of these vasculatures with a 5–0 nylon monofilament (Bear Medic Corporation, Ibaraki, Japan) (Fig. 2c and d). Finally, the skin was sutured.

Hair on all mice was clipped and a depilatory agent (VEET Hair Removal Cream; Reckitt Benckiser Japan, Tokyo, Japan) was used to remove residual hair before fluorescence imaging.

### Synthesis of DiR-modified liposomes

Egg phosphatidylcholine (EPC) and 1-(monomethoxy polyethylene glycol 2000)-2,3-dimyristoyl glycerol (DMG-PEG2k) were purchased from NOF Corporation (Kanagawa, Japan). Cholesterol (Chol) was purchased from Sigma (St. Louis, MO, USA). DiR was purchased from Promokine (Heidelberg, Germany).

DiR-modified liposomes were prepared using the lipid film hydration method. Briefly, a lipid film was prepared in a glass test tube by evaporation of a chloroform and ethanol solution of lipids, containing EPC, Chol, DMG-PEG2k, and DiR (molar ratio, 70:30:3:1; total lipid amount = 10 μmol). After preparation of the lipid film, it was hydrated with PBS for 10 min at room temperature. The total lipid concentration was 10 mM (EPC/Chol = 7/3 plus 3 mol% DMG-PEG2000 and 1 mol% DiR). After hydration, the lipid film was sonicated for 30 s at room temperature in a bath-type sonicator (AU-25C; Aiwa Co., Tokyo, Japan) to form complete liposomes. Finally, the liposome suspension was extruded through polycarbonate membrane filters (100-nm pore size; Nucleopore) with a Mini-extruder (Avanti Polar Lipids, Alabaster, AL, USA) to size the liposomes. The diameter and ζ-potential of the liposomes were determined using an electrophoretic light-scattering spectrophotometer (Zetasizer; Malvern Instruments, Worcestershire, UK). Physicochemical properties of DiR-labelled liposomes are shown in Table 1.

**Table 1:** Physicochemical properties of DiR-labelled liposomes. Mean ± SD of triplicate experiments.

	Size (d.nm)	ζ-potential (mV)	PdI
DiR-Liposome	114 ± 5	0.6 ± 0.1	0.08 ± 0.00

### Assessment of lymphatic flow at 1 week after resection of the popliteal LN

Ten ICR mice underwent resection of the popliteal LN. At day 7 after resection, they were evaluated using the PDE camera system. Their hair was clipped and residual hair was removed with the depilatory cream before imaging. Mice were anesthetised by isoflurane and injected with 0.02 ml DiR-modified liposomes into the left hind footpad. While mice were in the supine position, the course of lymphatic flow was investigated over time. Five ICR mice were also investigated in supine and dorsal positions at 12 weeks after resection.

### Analysis of the lymphatic transport of DiR-modified liposomes and ICG

To compare the lymphatic transport between DiR-modified liposomes and ICG (Diagnogreen; Daiichi Sankyo Company, Ltd., Tokyo, Japan), 0.02 ml of either DiR-modified liposomes or ICG (2.5 mg/ml) was injected into the footpad of each PLN-resected mouse, and time-dependent lymphatic transport was observed by the PDE.

### Determining the duration of the development of a novel route

To clarify when the novel route of lymphatic flow from the footpad to the ILN had developed, lymphatic flow was visualised by ICG injection at 1–3 days after the PLN resection using the PDE.

### Quantitative analysis

To conduct quantitative evaluations, we investigated linearity of the fluorescence intensity of DiR-labelled liposomes in the IVIS. DiR-labelled liposomes showed linearity in the fluorescence intensity range of 0.09–20.2 × 10^8^ photons/s (coefficient of determination = 0.99) (see Supplementary Fig. S1).

To measure the effect of PLN resection on changing the lymphatic route, fluorescent signals in LNs were compared between mice without any surgical treatment (normal) and mice at 1 week after PLN resection (PLN-resected) using the IVIS under the control of Living Image software (Caliper Life Science, Hopkinton, MA, USA) (n = 4 per group). Then, 0.02 ml of a DiR-modified liposome solution was injected into the footpad. At 120 min after the injection, mice were anaesthetised by 2% isoflurane, and abdominal skin was peeled from the epifascial layer of the abdominal muscles to reveal the LNs. ROIs were placed over the PLN, ILN, ALN, and liver, and total signal intensity values of each ROI were compared quantitatively. Imaging parameters included wavelengths ranging from 745 to 840 nm, an exposure time of 2 s; f/stop of 2; and field of view of 10.0 × 10.0 cm with medium binning.

To analyse the lymphatic transport of DiR-labelled liposomes, ROIs were placed at the ILN and ALN of PLN-resected mice. The fluorescent signals were sequentially monitored every 5 min using the IVIS after injection of liposomes into the footpad. The imaging parameters were fixed as described above. Injection sites were covered with rubber tape to block detection of saturated levels of fluorescent signals in this position.

### Evaluation of lymphatic flow change under lymphostasis

To analyse the change in lymphatic flow under lymphostasis, abdominal lymphedema was surgically induced in mice by PLN resection according to the method of Ogata *et al*.^17^. Then, at 1 week after PLN resection, skin was longitudinally incised along the midline of the abdomen, and the right abdominal skin was peeled from the epifascial layer of the abdominal muscles to expose the subdermal tissues including the ILN. EBD was then injected into the ILN to visualise the ALN and its collecting lymphatic vessels. Then, the ALN was resected and the collecting lymphatic vessels were ligated at approximately 5 mm caudal from the ALN. Finally, the skin was sutured to its original position (Fig. 6a). Quantitative analysis of the DiR-labelled liposomes was performed as described above at 8 weeks after the induction of abdominal lymphedema. ROIs were placed on the ILN and regions of abdominal lymphostasis (Fig. 6c).

### Histological and immunohistochemical analyses

To investigate whether PLN resection affected the abdominal lymphatic system, abdominal subcutaneous tissues were collected from PLN-resected mice (8 weeks after resection of the PLN) and normal mice as a negative control (Fig. 3c). For comparison, subcutaneous tissues of the lymphedema model established as described above were also examined (Fig. 6b). In this analysis, skin tissues located at 10 mm above the ILN along with the major collecting lymphatic vessels were resected. The tissues were fixed in 10% formalin and embedded in paraffin. The embedded specimens were sectioned (5 μm thicknesses, and stained with haematoxylin and eosin. Serial sections were deparaffinised and blocked with 2% bovine serum albumin for immunohistochemical staining. To identify lymphatic and blood vessels, a mouse anti-mouse actin α-smooth muscle (SMαA) antibody (A5228; Sigma-Aldrich, St Louis, MO, USA) and hamster anti-mouse podoplanin antibody (ab11936; Abcam PLC, Cambridge, UK) were used for double staining with corresponding secondary antibodies (anti-mouse from Dako, Glostrup, Denmark and anti-hamster from Jackson ImmunoResearch, Tokyo, Japan). SMαA was stained red using an ABC-alkaline phosphatase staining kit and Vector Red substrate (Vector Laboratories, Burlingame, CA, USA). After antigen retrieval by microwave treatment in citrate buffer at 95 °C for 20 min, podoplanin was stained brown using a streptavidin horseradish peroxidase reaction technique with diaminobenzidine (Dojindo Laboratories, Kumamoto, Japan).

### Statistical analysis

Data are shown the mean values ± standard error. Statistical analysis was performed by SPSS Version 20 (IBM Corp., Armonk, NY, USA). The Welch *t*-test was used to compare two groups. P < 0.05 was considered as statistically significant.

### Data availability

The datasets generated and/or analysed in the current study are available from the corresponding author upon reasonable request.

## Electronic supplementary material

Supplementary Information

## References

[1] Karpanen T, Alitalo K (2008). Molecular biology and pathology of lymphangiogenesis. Annu. Rev. Pathol..

[2] Jurisic G, Detmar M (2009). Lymphatic endothelium in health and disease. Cell Tissue Res..

[3] Frangioni JV (2003). *In vivo* near-infrared fluorescence imaging. Curr. Opin. Chem. Biol..

[4] Rasmussen JC, Tan IC, Marshall MV, Fife CE, Sevick-Muraca EM (2009). Lymphatic imaging in humans with near-infrared fluorescence. Curr. Opin. Biotechnol..

[5] Proulx ST (2010). Quantitative imaging of lymphatic function with liposomal indocyanine green. Cancer Res..

[6] Trevaskis NL, Kaminskas LM, Porter CJ (2015). From sewer to saviour - targeting the lymphatic system to promote drug exposure and activity. Nat. Rev. Drug Discov..

[7] Van den Broeck W, Derore A, Simoens P (2006). Anatomy and nomenclature of murine lymph nodes: Descriptive study and nomenclatory standardization in BALB/cAnNCrl mice. J. Immunol. Methods..

[8] Hayashi K (2007). Real-time imaging of tumor-cell shedding and trafficking in lymphatic channels. Cancer Res..

[9] Kodama T, Hatakeyama Y, Kato S, Mori S (2014). Visualization of fluid drainage pathways in lymphatic vessels and lymph nodes using a mouse model to test a lymphatic drug delivery system. Biomed. Opt. Express..

[10] Ruddell A (2008). Dynamic contrast-enhanced magnetic resonance imaging of tumor-induced lymph flow. Neoplasia..

[11] Kwon S, Sevick-Muraca EM (2010). Functional lymphatic imaging in tumor-bearing mice. J. Immunol. Methods..

[12] Kwon S, Sevick-Muraca EM (2011). Mouse phenotyping wit near-infrared fluorescence lymphatic imaging. Biomed Opt Express..

[13] Long KM, Heise M (2013). Safe and effective mouse footpad inoculation. Methods Mol. Biol..

[14] Hoshi H, Kamiya K, Endo E (1981). Cortical structure of the lymph node. I. Effect of blockage of the afferent lymph flow to mouse popliteal nodes for protracted periods. J. Anat..

[15] Tilney NL (1970). The systemic distribution of soluble antigen injected into the footpad of the laboratory rat. Immunology..

[16] Bagby TR (2012). Lymphatic trafficking kinetics and near-infrared imaging using star polymerarchitectures with controlled anionic character. Eur. J. Pharm. Sci..

[17] Kwon S, Agollah GD, Wu G, Sevick-Muraca EM (2014). Spatio-temporal changes of lymphatic contractility and drainage patterns following lymphadenectomy in mice. PLoS One..

[18] Ogata F (2016). Excess Lymphangiogenesis Cooperatively Induced by Macrophages and CD4(+) T Cells Drives the Pathogenesis of Lymphedema. J. Invest. Dermatol..

[19] Robinson HA (2013). Non-invasive optical imaging of the lymphatic vasculature of a mouse. J. Vis. Exp..

[20] Miotti R (1965). The lymph nodes and lymph vessels of the white rat (Rattus norvegicus Berkenhaut, Epimys norvegicus). Acta. Anat. (Basel)..

[21] Tilney NL (1971). Patterns of lymphatic drainage in the adult laboratory rat. J. Anat..

[22] Rutkowski JM, Moya M, Johannes J, Goldman J, Swartz MA (2006). Secondary lymphedema in the mouse tail: Lymphatic hyperplasia, VEGF-C upregulation, and the protective role of MMP-9. Microvasc. Res..

[23] Jitariu AA, Cimpean AM, Kundnani NR, Raica M (2015). Platelet-derived growth factors induced lymphangiogenesis: evidence, unanswered questions and upcoming challenges. Arch. Med. Sci..

[24] Uenosono Y (2003). Evaluation of colloid size for sentinel nodes detection using radioisotope in early gastric cancer. Cancer Lett..

[25] Nakajima M, Takeda M, Kobayashi M, Suzuki S, Ohuchi N (2005). Nano-sized fluorescent particles as new tracers for sentinel node detection: experimental model for decision of appropriate size and wavelength. Cancer Sci..

[26] Leidenius MH, Leppänen EA, Krogerus LA (2004). & Smitten, K. A. The impact of radiopharmaceutical particle size on the visualization and identification of sentinel nodes in breast cancer. Nucl. Med. Commun..

[27] Zhang Y, Toyota T, Matsubara H, Hayashi H (2017). Biodistribution of liposomes in the lymphatics according to particle size. Japanese Journal of Lymphology..

[28] Weissleder R, Thrall JH (1989). The lymphatic system: diagnostic imaging studies. Radiology..

[29] Henze E (1982). Lymphoscintigraphy with Tc-99m-labeled dextran. J. Nucl. Med..

[30] Barrett T, Choyke PL, Kobayashi H (2006). Imaging of the lymphatic system: new horizons. Contrast Media Mol. Imaging..

[31] Oussoren C, Zuidema J, Crommelink DJ, Storm G (1997). Lymphatic uptake and biodistribution of liposomes after subcutaneous injection. II. Influence of liposomal size, lipid composition and lipid dose. Biochim. Biophys. Acta..

[32] Gardenier JC (2016). Diphtheria toxin-mediated ablation of lymphatic endothelial cells results in progressive lymphedema. JCI Insight..

