# Supplementary material for: Development of a mouse model for the visual and quantitative assessment of lymphatic trafficking and function by in vivo imaging
Source: Sci Rep. 2018 Apr 12;8:5921. doi: 10.1038/s41598-018-23693-9 (PMC5897414; doi:10.1038/s41598-018-23693-9)
Supplement: Supplementary file 1 — Supplementary Information [file 41598_2018_23693_MOESM1_ESM.pdf]

Supplementary Information for

Development of a mouse model for the visual and quantitative assessment of lymphatic trafficking and function by *in vivo* imaging

Yoshihisa Yamaji[1], Shinsuke Akita[1], Hidetaka Akita[2], Naoya Miura[2], Masaki Gomi[2], Ichiro Manabe[3], Yoshitaka Kubota[1] and Nobuyuki Mitsukawa[1,*]

[1]Department of Plastic, Reconstructive, and Aesthetic Surgery, Chiba University Graduate School of Medicine, Chiba 2608677, Japan

[2]Laboratory of Pharmacology and Toxicology, Chiba University Graduate School of Pharmaceutical Sciences, Chiba 2608677, Japan

[3]Department of Aging Research, Chiba University Graduate School of Medicine, Chiba 2608677, Japan

*Corresponding author: Nobuyuki Mitsukawa, MD, PhD

Department of Plastic, Reconstructive, and Aesthetic Surgery, Chiba University

Graduate School of Medicine, Chiba 2608677, Japan

Tel: +81-43-222-7171

Fax: +81-43-226-2316

E-mail: nmitsu@air.linkclub.or.jp

This supplement contains:

Supplementary Figure S1

Supplementary Fig. S1. Linearity between fluoresce signals in IVIS imaging and the lipid concentration.

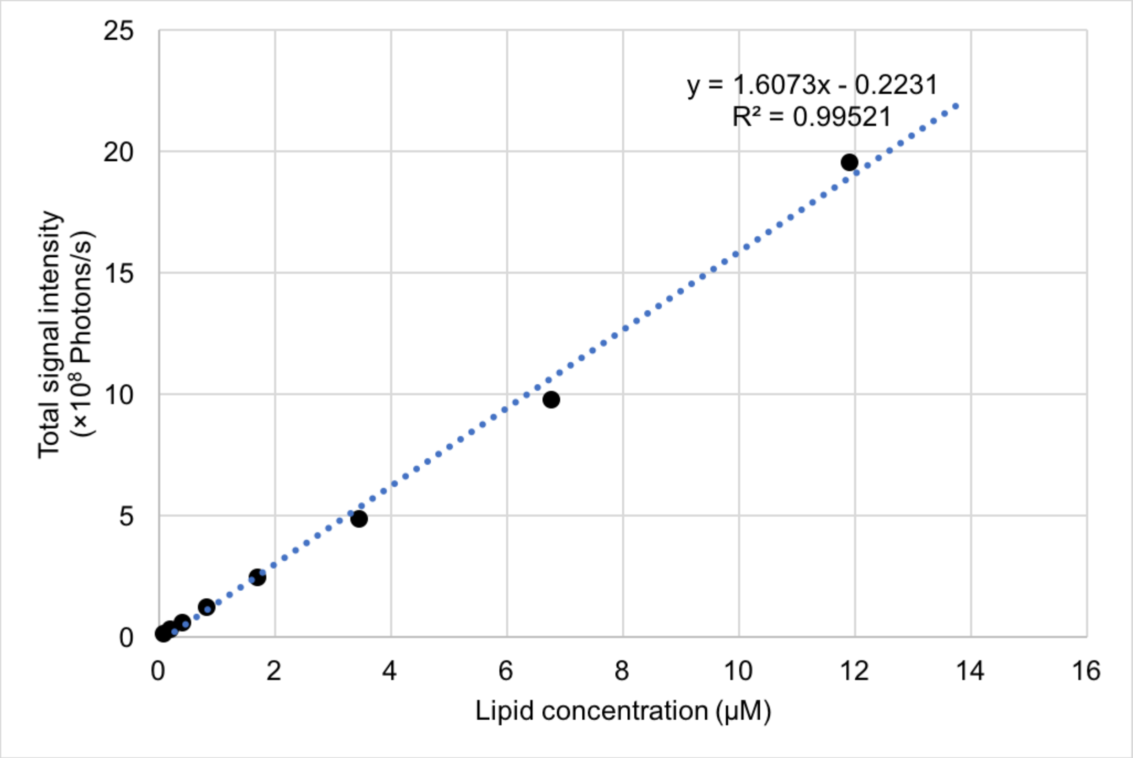

Aliquots (100 μL) of DiR-labelled liposome solutions in a series of dilutions were applied to a 96-well plate (total lipid concentrations: 0.15–156 μM) and then subjected to IVIS imaging using the same settings as those in the animal experiments. The fluorescent signals in photons/s were increased linearly depending on the concentration of liposomes. Of note, the maximum intensity of the fluorescent signals in animal experiments were within this linear range.
